# Effect of Different Molecular Weights of Polyacrylic Acid on Rat Lung Following Intratracheal Instillation

**DOI:** 10.3390/ijms231810345

**Published:** 2022-09-07

**Authors:** Chinatsu Nishida, Hiroto Izumi, Taisuke Tomonaga, Ke-Yong Wang, Hidenori Higashi, Jun-Ichi Takeshita, Ryohei Ono, Kazuki Sumiya, Shota Fujii, Yuki Hata, Kazuo Sakurai, Yasuyuki Higashi, Kei Yamasaki, Kazuhiro Yatera, Yasuo Morimoto

**Affiliations:** 1 Department of Respiratory Medicine, University of Occupational and Environmental Health, Japan 1-1 Iseigaoka, Yahata-nishi-ku, Kitakyushu 807-8555, Fukuoka, Japan; 2 Department of Occupational Pneumology, Institute of Industrial Ecological Sciences, University of Occupational and Environmental Health, Japan 1-1 Iseigaoka, Yahata-nishi-ku, Kitakyushu 807-8555, Fukuoka, Japan; 3 Shared-Use Research Center, School of Medicine, University of Occupational and Environmental Health, Japan 1-1 Iseigaoka, Yahata-nishi-ku, Kitakyushu 807-8555, Fukuoka, Japan; 4 Department of Environmental Health Engineering, Institute of Industrial Ecological Sciences, University of Occupational and Environmental Health, Japan 1-1 Iseigaoka, Yahata-nishi-ku, Kitakyushu 807-8555, Fukuoka, Japan; 5 Research Institute of Science for Safety and Sustainability, National Institute of Advanced Industrial Science and Technology (AIST), Tsukuba, Japan 16-1 Onogawa, Tsukuba 305-8569, Ibaraki, Japan; 6 Department of Chemistry and Biochemistry, The University of Kitakyushu, 1-1, Hibikino, Wakamatsu-ku, Kitakyushu 808-0135, Fukuoka, Japan

**Keywords:** polyacrylic acid (PAA), organic compounds, molecular weight, pulmonary toxicity

## Abstract

Background: We conducted intratracheal instillations of different molecular weights of polyacrylic acid (PAA) into rats in order to examine what kinds of physicochemical characteristics of acrylic acid-based polymer affect responses in the lung. Methods: F344 rats were intratracheally exposed to a high molecular weight (HMW) of 598 thousand g/mol or a low molecular weight (LMW) of 30.9 thousand g/mol PAA at low and high doses. Rats were sacrificed at 3 days, 1 week, 1 month, 3 months and 6 months post exposure. Results: HMW PAA caused persistent increases in neutrophil influx, cytokine-induced neutrophil chemoattractants (CINC) in the bronchoalveolar lavage fluid (BALF), and heme oxygenase-1 (HO-1) in the lung tissue from 3 days to 3 months and 6 months following instillation. On the other hand, LMW PAA caused only transient increases in neutrophil influx, CINC in BALF, and HO-1 in the lung tissue from 3 days to up to 1 week or 1 month following instillation. Histopathological findings of the lungs demonstrated that the extensive inflammation and fibrotic changes caused by the HMW PAA was greater than that in exposure to the LMW PAA during the observation period. Conclusion: HMW PAA induced persistence of lung disorder, suggesting that molecular weight is a physicochemical characteristic of PAA-induced lung disorder.

## 1. Introduction

It is thought that organic compounds do not induce irreversible interstitial lesions such as pulmonary fibrosis, unlike inorganic compounds such as asbestos and crystalline silica, but pulmonary fibrosis caused by organic compounds has been reported recently. Polyhexamethyleneguanidine phosphate (PHMG-p), an organic compound used as a humidifier disinfectant, was reported to cause lung disorders, including interstitial lung disease, emphysema and acute respiratory distress syndrome (ARDS), in 5955 people in South Korea [1,2]. In Japan, it was reported that in a workplace where cross-linked acrylic acid-based polymers (CL-PAA) were handled, six out of tens of workers who had used a powder made of the polymers developed progressive lung disorders such as pneumoconiosis, emphysema and pneumothorax [3]. Intratracheal exposure to CL-PAA has also been reported to induce pulmonary inflammation in rat lung as severe or equivalent to crystalline silica and asbestos exposure [4]. It was confirmed from the above that CL-PAA, which is an organic compound, has severe inflammatory and fibrotic potentials in the lung. It is unclear, however, what physicochemical characteristics of CL-PAA are closely related to lung disorder. 

Acrylic acid-based polymers show different functions depending on their molecular weight, and as the molecular weight increases, the functions change in the order of dispersion, thickening, aggregation, and swelling [5]. Thickening contributes to the delay in clearance of the substance from the lungs [6], it is, therefore, considered that the molecular weight is involved in lung disorders caused by polyacrylic acid (PAA).

The mechanism of lung disorder caused by organic compounds is unknown, but, with inorganic compounds, the inhaled chemicals deposit in the lung, causing persistent inflammation and eventually leading to the formation of chronic and irreversible lesions such as pulmonary fibrosis and tumors [7,8,9,10,11]. Asbestos, crystalline silica, and nanoparticles with high pulmonary toxicity have been reported to cause persistent inflammation in the lungs, leading to lung fibrosis and tumorigenesis [12,13]. It is, therefore, presumed that persistent lung inflammation is a crucial process in the induction and progression of chronic and irreversible lung lesions in lung disorder caused by inhalable dust [7,9,10,11].

We conducted intratracheal instillations of different molecular weights into the lungs of rats in order to explore whether or not molecular weight, among the physicochemical characteristics of PAA, which is the basic structure of CL-PAA, affects responses in the lung. We used high and low molecular weights of PAA (HMW PAA and LMW PAA) and examined pulmonary inflammation and fibrosis.

## 2. Results

### 2.1. Characterization of PAA

The fundamental characteristics of the HMW and the LMW PAA are summarized in Table 1.

In summary, the polymers used in our study had weight average molecular weights (*M*_W_) of 598 thousand g/mol (HMW) and 30.9 thousand g/mol (LMW), respectively. The number average molecular weights (Mn) of HMW and LMW were 451 thousand and 25.8 thousand, respectively. The poly dispersity indexes (PDIs) were 1.33 (HMW) and 1.20 (LMW), respectively. The radii of gyration (Rg) of the polymers in the suspension were 57.5 nm (HMW) and 13.5 nm (LMW), respectively. The hydrodynamic radii (Rh) of the polymers in the molecular dispersion were 39.7 nm (HMW) and 2.78 nm (LMV), respectively. The hydrodynamic radii (Rh) of the polymers in the H_2_O dispersion were 217 nm (HMW) and 1.11 nm (LMV), respectively. The secondary diameters in the bulk were 2.83 µm (HMW) and 269 nm (LMW), respectively. The secondary diameters in the suspension were 39.3 mm (HMW) and 53.2 nm (LMW), respectively. 

**Figure 1:**
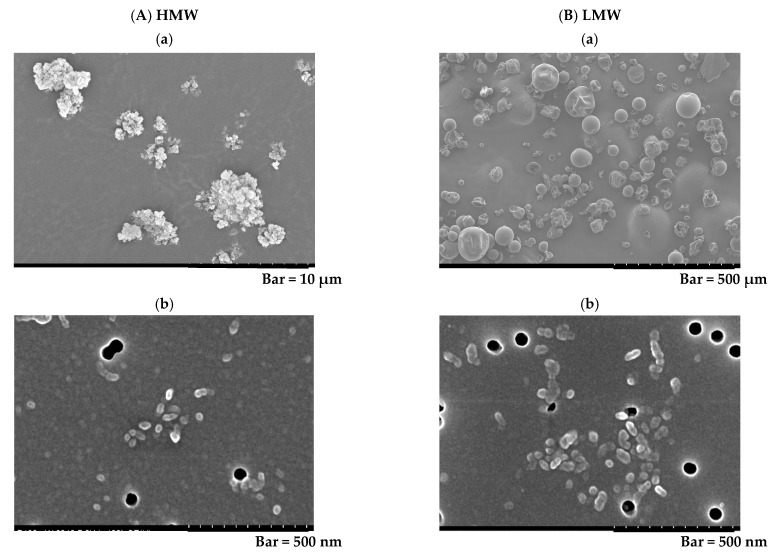
Scanning electron microscopy (SEM) images of the polymers used in the present study. The bulk HMW and LMW PAA powders and the suspended HMW and LMW PAA in distilled water are shown in (**A**(**a**)) and (**B**(**a**)), and (**A**(**b**)) and (**B**(**b**)), respectively. They all formed agglomerates. (Internal scale bar = 10 μm for (**A**(**a**)), 500 nm for (**A**(**b**)), 500 µm for (**B**(**a**)) and 500 nm for (**B**(**b**))).

Figure 1 shows that the bulk polymers (Figure 1A(a): HMW PAA, and Figure 1B(a): LMW PAA) and the polymers in the solution formed agglomerates (Figure 1A(b): HMW PAA, and Figure 1B(b): LMW PAA).

No endotoxin was detected in any of the polymer suspensions.

### 2.2. Relative Lung Weights

**Figure 2:**
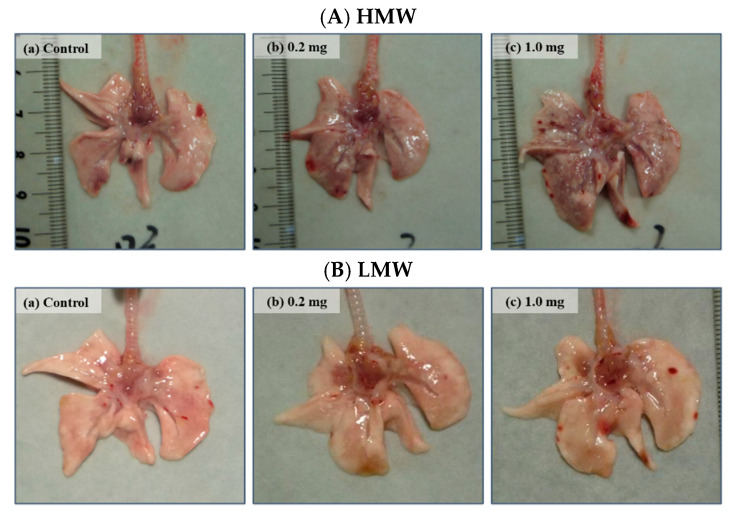
Macro findings at 3 days after instillation. The lungs in the exposed groups showed swelling at 3 days after intratracheal instillation. (**A**) HMW PAA-exposure group and (**B**) LMW PAA-exposure group, where (**a**) control group, (**b**) 0.2 mg-exposure group, and (**c**) 1.0 mg-exposure group. In the HMW PAA group, the surface of the lungs were rough.

Relative lung weight (lung weight/body weight) increased dose dependently at each observation point in both the HMW and LMW PAA-exposure groups. In the HMW PAA-exposure group; in particular, a significant increase in relative lung weight was sustained throughout the observation period compared to the control group (Table 2). The HMW PAA-exposure lungs, especially the 1.0 mg-HMW PAA-exposure lungs, were more edematous and mottled than the LMW PAA-exposure lungs at 3 days after the instillation (Figure 2A(a–c),B(a–c)).

### 2.3. Cell Analysis and Lactate Dehydrogenase (LDH) Activity in Bronchoalveolar Lavage Fluid (BALF)

**Figure 3:**
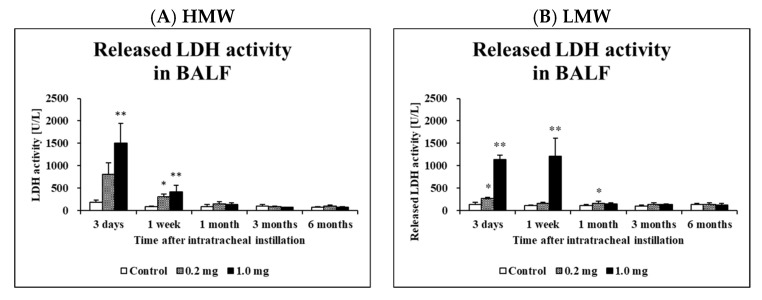
Analysis of released LDH activity in BALF following intratracheal instillation. (**A**) HMW PAA-exposure group and (**B**) LMW PAA-exposure group. Released LDH activity in BALF in all of the exposed groups were higher than those in the control groups in a dose dependent-manner. Data are presented as mean ± SE. (* *p* < 0.05, ** *p* < 0.01).

**Figure 4:**
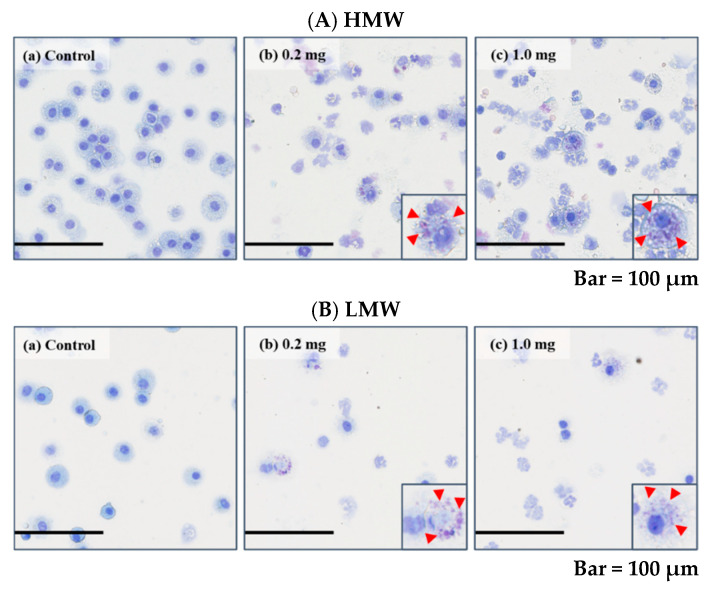
Cells in BALF at 3 days after the instillation. The images of cells in BALF are shown in Figure 4. (**A**) HMW PAA-exposure group and (**B**) LMW PAA-exposure group, where (**a**) control group, (**b**) 0.2 mg-exposure group, and (**c**) 1.0 mg-exposure group. Polymer phagocytosed macrophages are shown in the insets (red arrow heads). (internal scale bar = 100 μm for all).

Table 3 and Figure 3 shows the results of inflammatory cell counts and LDH activity, an index of cell injury, in the BALF. A significant increase in total cell count was observed in the HMW PAA-exposure group at 3 days to 1 month after exposure at both the 0.2 mg and 1.0 mg doses compared to the control group, whereas it was observed in the LMW PAA-exposure group at 3 days to 1 week after exposure at both or either the 0.2 mg and 1.0 mg dose compared to the control group. There were significant increases in the number of macrophages and neutrophils, and the percentage of neutrophils from 3 days to 1 month after exposure in the HMW PAA-exposure group, and from 3 days to 1 week after exposure in the LMW PAA-exposure group (Table 3). Many neutrophils and many macrophage-phagocytosed PAA in the exposure groups could be seen in light microscopic images of the BALF at 3 days after the instillation (Figure 4A(a–c),B(a–c)), indicating that PAA induced lung inflammation. The results of released LDH activity in the 0.2 mg and 1.0 mg-HMW PAA exposure groups also showed statistically significant increases at 1 week and from 3 days to 1 week after exposure compared to the control group (Figure 3A), whereas the results in the 0.2 mg and 1.0 mg LMW PAA-exposure groups showed statistically significant increases from 3 days to 1 week or 1 month after exposure compared to the control group (Figure 3B).

### 2.4. Concentration of Cytokine-Induced Neutrophil Chemoattractants (CINC) in BALF and Concentration of Heme Oxygenase (HO)-1 in Lung Tissue

**Figure 5:**
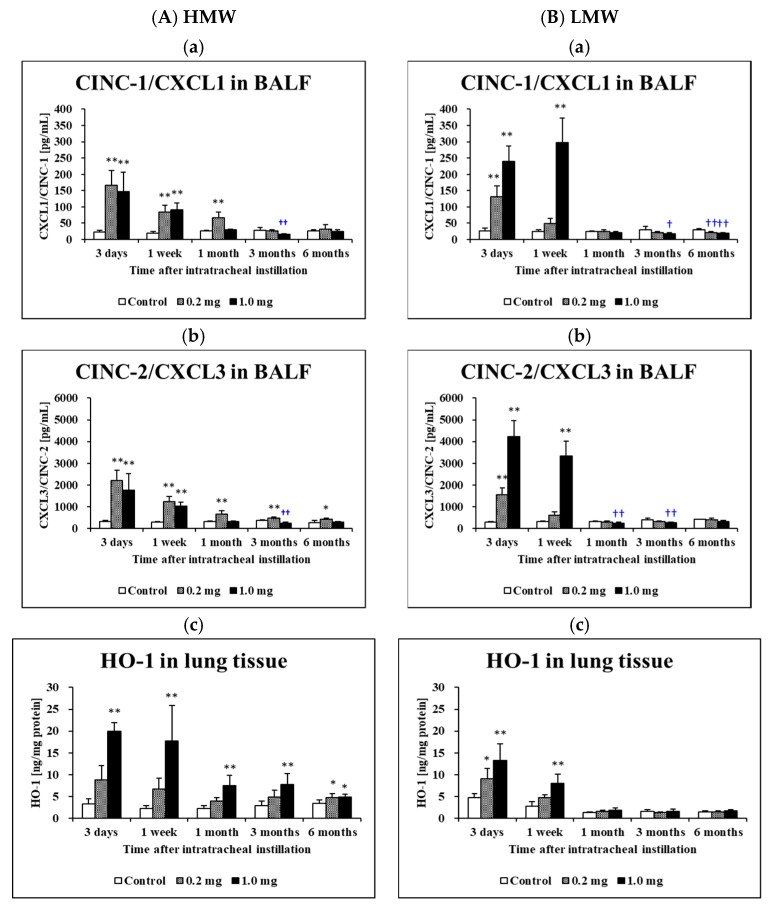
Analysis of cytokines in BALF and HO-1 in lung tissue after intratracheal instillation (**A**) HMW PAA-exposure group and (**B**) LMW PAA-exposure group, where (**a**) CINC-1/CXCL1 concentration in BALF, (**b**) CINC-2/CXCL3 concentration in BALF, and (**c**) HO-1 concentration in lung tissue. In the HMW PAA-exposure group, high expression of CINC-1/CXCL1 and CINC-2/CXCL3 in BALF was persistent from 3 days to 1 or 3 months after the instillation, whereas in the LMW PAA-exposure group, it was persistently only from 3 days to 1 week after the instillation. In the HMW PAA-exposure group, high expression of HO-1 was sustained throughout the observation period, but in the LMW PAA-exposure group it was sustained for only 1 week after the instillation. Data are presented as mean ± SE. (* *p* < 0.05 and ** *p* < 0.01 indicate that the values are significantly higher than control group. † *p* < 0.05 and †† *p* < 0.01 indicate that the values are significantly lower than control group.).

Figure 5 shows the concentrations of CINC-1 (Figure 5A(a),B(a)) and CINC-2 (Figure 5A(b),B(b)) in the BALF following the intratracheal instillation of the HMW PAA and LMW PAA, respectively. The concentrations of CINC-1 increased persistently from 3 days until 1 month post exposure in the HMW PAA-exposure group and from 3 days until 1 week post exposure in the LMW PAA-exposure group compared to their respective control groups. The concentrations of CINC-2 increased persistently from 3 days until 6 months post exposure in the HMW PAA-exposure group and from 3 days until 1 week post exposure in the LMW PAA-exposure group compared to their respective control groups. The expression levels of these chemokines in the exposed groups generally decreased over time. Statistically significant persistent increases in the concentration of HO-1 in the lung tissues exposed to the HMW PPA were observed from 3 days to 6 months, whereas in the lung tissues exposed to the LMW PPA, there was only a transient increase (Figure 5A(c),B(c)).

### 2.5. Micro-CT Imaging

**Figure 6:**
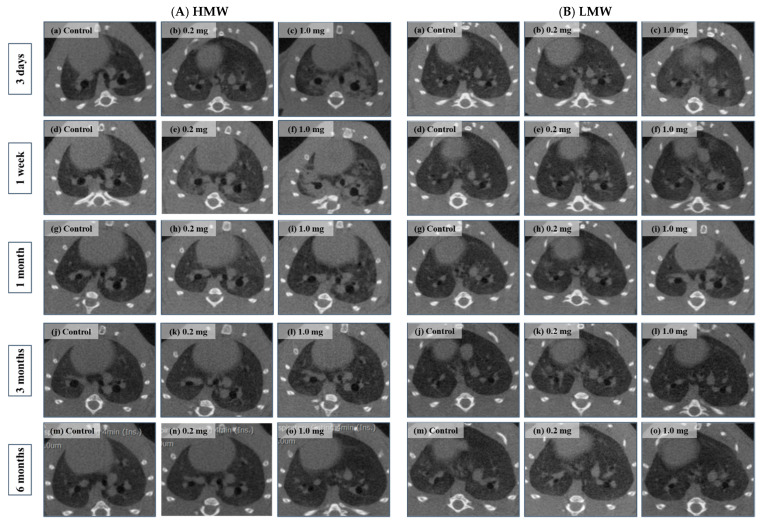
3D micro-CT imaging after intratracheal instillation. (**A**) HMW PAA-exposure group and (**B**) LMW PAA-exposure group at 3 days, where (**a**) control group, (**b**) 0.2 mg-exposure group, and (**c**) 1.0 mg-exposure group; at 1 week, where (**d**) control group, (**e**) 0.2 mg-exposure group, and (**f**) 1.0 mg-exposure group; at 1 month, where (**g**) control group, (**h**) 0.2 mg-exposure group, and (**i**) 1.0 mg-exposure group; at 3 months, where (**j**) control group, (**k**) 0.2 mg-exposure group, and (**l**) 1.0 mg-exposure group; and at 6 months, where (**m**) control group, (**n**) 0.2 mg-exposure group, and (**o**) 1.0 mg-exposure group. Bilateral diffusion or centrilobular infiltration in lungs was observed from 3 days to 1 month in the HMW PAA-exposure group and from 3 days to 1 week or 1 month in the LMW PAA-exposure group, respectively. The radiological findings were more prominent in the HMW PAA-exposure group than in the LMW PAA-exposure group.

Diffuse or centrilobular infiltration was revealed in the lungs from 3 days to 1 month after exposure in the HMW PAA-exposure group and from 3 days to 1 week after exposure in the LMW PAA-exposure group, respectively, in a dose-dependent manner. Lung infiltration improved over time after 3 months in the HMW PAA-exposure group, and after 1 month in the LMW PAA-exposure group (Figure 6A(a–o),B(a–o)).

### 2.6. Histopathological Features in the Lung 

**Figure 7:**
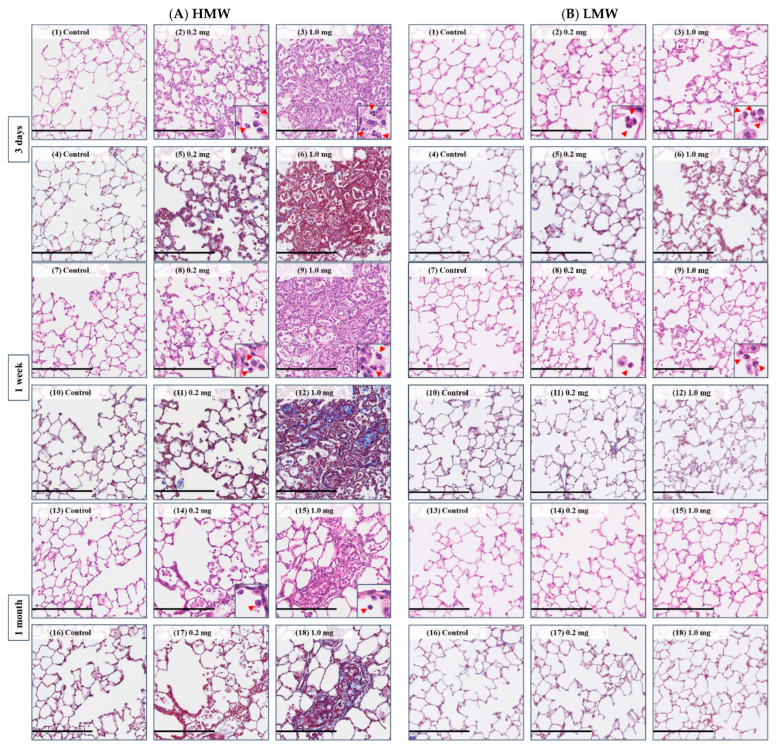

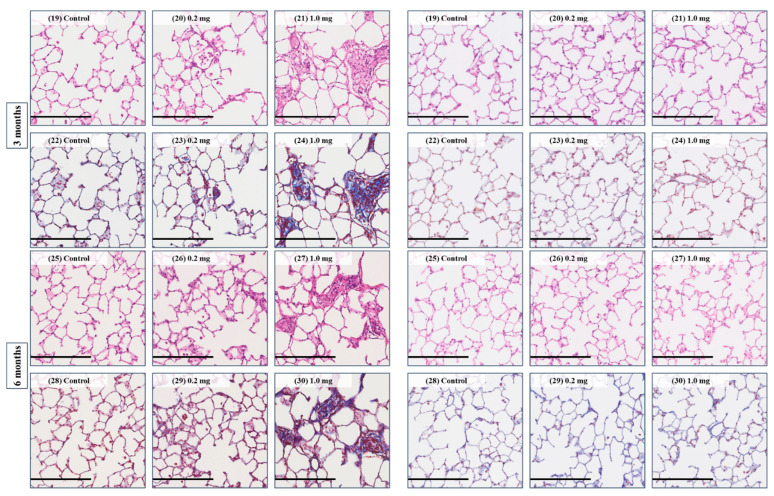
Comparison of histological findings between HMW PAA-exposure group and LMW PAA-exposure group (Hematoxylin and eosin (HE) and Masson trichrome (MT) staining). (**A**) HMW PAA-exposure group and (**B**) LMW PAA-exposure group. At 3 days HE staining, where (**1**) control group, (**2**) 0.2 mg-exposure group, and (**3**) 1.0 mg-exposure group; and MT staining, where (**4**) control group, (**5**) 0.2 mg-exposure group, and (**6**) 1.0 mg-exposure group. At 1 week HE staining, where (**7**) control group, (**8**) 0.2 mg-exposure group, and (**9**) 1.0 mg-exposure group; and MT staining, where (**10**) control group, (**11**) 0.2 mg-exposure group, and (**12**) 1.0 mg-exposure group. At 1 month HE staining, where (**13**) control group, (**14**) 0.2 mg-exposure group, and (**15**) 1.0 mg-exposure group; and MT staining, where (**16**) control group, (**17**) 0.2 mg-exposure group, and (**18**) 1.0 mg-exposure group. At 3 months HE staining, where (**19**) control group, (**20**) 0.2 mg-exposure group, and (**21**) 1.0 mg-exposure group; and MT staining, where (**22**) control group, (**23**) 0.2 mg-exposure group, and (**24**) 1.0 mg-exposure group. At 6 months HE staining, where (**25**) control group, (**26**) 0.2 mg-exposure group, (**27**) 1.0 mg-exposure group. MT staining; (**28**) control group, (**29**) 0.2 mg-exposure group, and (**30**) 1.0 mg-exposure group. In the HMW PAA-exposure group, lung inflammation, including infiltration of neutrophils in alveoli (red arrow heads in inset), persisted for 1 month, and pulmonary fibrosis observed after 3 days after the instillation was observed throughout the observation period. In the LMW PAA-exposure group, on the other hand, lung inflammation lasted only for 1 week or 1 month, and slight pulmonary fibrosis was observed at 3 days after the instillation, although it disappeared by 3 months. The extent of lung inflammation and fibrosis were more severe in the HMW PAA-exposure group than in the LMW PAA-exposure group. (Internal scale bar = 250 μm for all).

**Figure 8:**
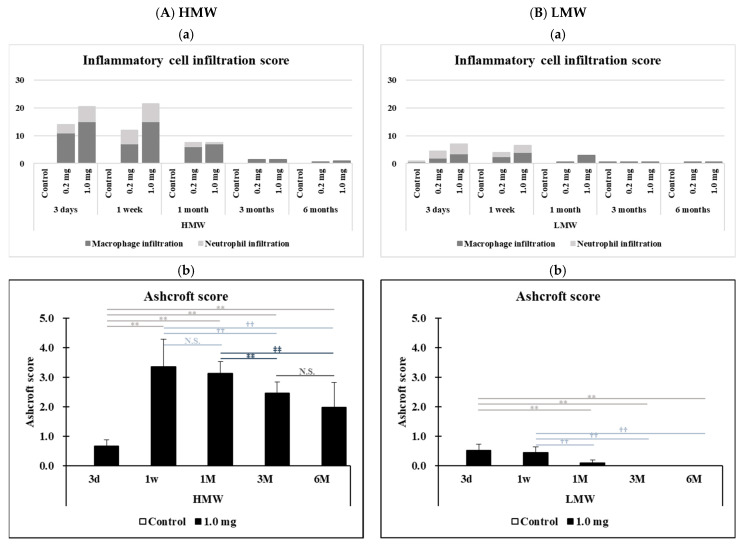
The histological evaluation of lung lesions. (**A**) HMW PAA-exposure group and (**B**) LMW PAA-exposure group, where (**a**) inflammatory cell infiltration score among the control, 0.2 mg-exposed, and 1.0 mg-exposed groups, and (**b**) Ashcroft score in the control group and 1.0 mg-exposure group. Inflammatory cell infiltration was observed in a dose-dependent manner, and was more severe and lasted longer in the HMW PAA-exposure group than in the LMW PAA-exposure group (**A**(**a**),**B**(**a**)). Pulmonary fibrosis was seen throughout the observation period in the HMW PAA-1.0 mg exposure group, whereas it was transient in the LMW PAA-1.0 mg exposure group. Pulmonary fibrosis was more severe in the HMW PAA-exposure group than in the LMW PAA-exposure group (**A**(**b**),**B**(**b**)). Data are presented as mean ± SE. (** *p* < 0.01, †† *p* < 0.01 and ‡‡ *p* < 0.01 indicate that there is a significant difference in the scores between the two groups, respectively. N.S. indicates that there is no significant difference in the scores between the two groups.).

Figure 7A(1–30),B(1–30) show representative histopathological findings in the lung in the HMW PAA-exposure group and the LMW PAA-exposure group, respectively, during the observation period. Inflammatory cell infiltrations, mainly macrophages and neutrophils, into the alveoli were seen in a dose-dependent manner in both the HMW and LMW PAA-exposure groups. Lung inflammation in histopathology was more marked in the HMW PAA-exposure group than in the LMW PAA-exposure group, and although it persisted even at 1 month after exposure in the HMW PAA-exposure group (Figure 7A(1–3,7–9,13–15,19–21,25–27) and Figure 8A(a)), it was only transient in the LMW PAA-exposure group (Figure 7B(1–3,7–9,13–15,19–21,25–27) and Figure 8B(a)). There were no granulomas or formation of giant cells in any of the groups. 

Pulmonary fibrosis was more severe in the HMW PAA-exposure group than in the LMW PAA-exposure group in general. In addition, while the pulmonary fibrosis was observed in the HMW PAA-exposure lung throughout the observation period, it was transient in the LMW PAA-exposure lung (Figure 7A(4–6,10–12,16–18,22–24,28–30),B(4–6,10–12,16–18,22–24,28–30) and Figure 8A(b),B(b) and Table A1). 

## 3. Discussion

The main findings obtained in the present study are as follows: (1) the HMW PAA caused severe and persistent lung inflammation and fibrosis compared to the LMW; and (2) the HO-1 protein level in the lung tissue exposed to the HMW PAA increased persistently during the observation period compared to the LMW.

In the present study, significant and persistent neutrophil-based inflammatory cell infiltration in the alveoli was observed in the lungs, especially following intratracheal instillation of PAA with HMW. In particular, the severity and persistence of the lung inflammation due to PAA was comparable to or greater than that of inorganic compounds with high toxicity. 

We previously conducted intratracheal instillations of various inorganic compounds such as crystalline silica, asbestos (chrysotile), and nanoparticles of nickel oxide (NiO) [14] and cerium oxide (CeO_2_) [15], and multi-walled carbon nanotube (MWCNT) [16] under the same experimental conditions of dose and observation period. Although these inorganic materials also induced persistent neutrophil-based inflammatory cell infiltration in the alveoli, the lung inflammation caused by HMW PAA was equal to or greater than that of those materials. 

Concerning lung disorder induced by other organic compounds, there have been some reports from South Korea of intratracheal instillation and inhalation exposure to PHMG-P in animal models, and severe lung inflammation, mainly neutrophils, was observed in all of these studies [17,18]. The clinical features of PHMG-P-caused lung disorder in humans are a short latent period before the onset of lung disorder, rapid development of fibrosis following severe pneumonia, and a high mortality rate [19,20]. It is possible that organic compounds have different physicochemical characteristics than inorganic compounds, and new substances that have potential for serious lung injury may emerge in the future. 

It has also been reported in occupational asthma that the effect of substances on the lungs differs depending on the difference in molecular weight [21,22]. Occupational asthma due to HMW agents, grain flours such as wheat and buckwheat flour, wood dust, and pollen, is related to occupational rhinitis, conjunctivitis, atopy, and immediate asthmatic response, while occupational asthma due to LMW agents, isocyanates, polymers, and cosmetics involved in manufacturing and painting is related to chest discomfort and sputum during work, late asthmatic response, and acute severe asthma exacerbation. It is considered that the difference in molecular weight of sensitizers that cause occupational asthma involves allergic responses that are associated with immunoglobulin E (IgE) or less associated with IgE [23]. Compared to occupational asthma, in which the difference in the molecular weight of the sensitizer affects its phenotype, the difference in the molecular weight of PAA does not affect its phenotype of inflammation and is considered to affect the severity and range of inflammation in PAA-induced lung disorder.

Acrylic acid-based polymers, including PAA, change their function in the order of dispersion, thickening, aggregation, and swelling as the molecular weight increases [5]. Dispersion is related to the ion capturing function of the polymers [24]. Thickening is based on interactions, such as hydrogen bonds, ionic bonds, and hydrophobic bonds, between the polymers and the solvents [5]. Aggregation is due to the formation of crosslinking between the polymers and the dispersoids and the neutralization of the surface charge of the dispersoid by electrolytic groups [25,26,27]. Swelling is affected by the difference in osmotic pressure between the inside and outside of the polymers that occurs when the polymer comes into contact with the dispersion medium, the hydrophilicity of the polymers, and the crosslink density [5]. Melt viscosity of entangled linear polymers has been described to be proportional to their molecular weight to the 3.4th power [28,29], and the thickening that comes with increasing molecular weight affects the delay in clearance of the compounds from the lungs. It has been reported that the rate of mucus movement due to coughing is inversely related to the viscosity of the mucus [6,30]. If a highly viscous substance is present in the lungs, it is difficult for it to be excreted, which can lead to lung disorder. Cystic fibrosis (CF) is a recessive genetic disease that is characterized by airway mucus plugging and reduced mucus clearance, and is a disease derived from atelectasis and luminal obstruction to lung fibrosis and respiratory failure through infection [31,32]. 

**Figure 9:**
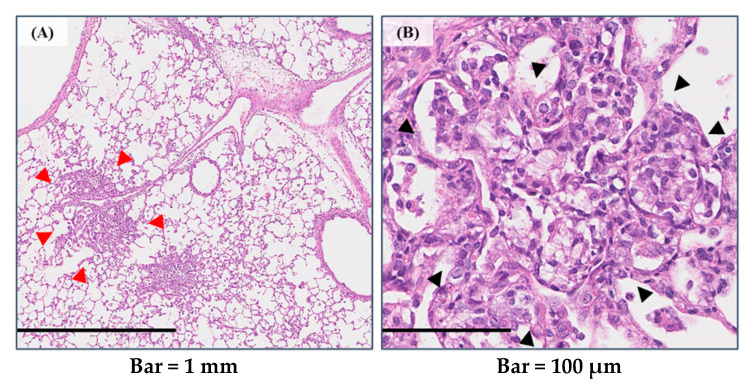
Distribution of lung inflammation in the exposed group. Peribronchiolar pulmonary inflammation was found (red arrow heads), and the alveolar spaces were filled with inflammatory cells (black arrow heads) (in HMW PAA-exposure group). (**A**) Low-power field; (**B**) high-power field. (Internal scale bar = 1 mm for (**A**) and 100 μm for (**B**)).

In the present study, we think that the strong viscosity of the injection solution by PAA enhanced the severity and range of inflammation. Inflammation in the pathological lung tissue was observed mainly in the peripheral airways, and atelectasis was observed in the surrounding alveolar space (Figure 9A,B). There are case reports in which treatment to reduce the viscosity of sputum was effective for atelectasis. For instance, it was reported that bronchoscopic instillation of DNase into atelectatic lobes resulted in a significant response in all of three children with CF in the following days, with further improvement up to four weeks after instillation [33]. A meta-analysis of 402 pediatric patients with non-CF pulmonary atelectasis also showed benefits in patients who received DNase for refractory atelectasis [34]. Based on these results, we believe that the viscosity of PAA and lung inflammation are closely related. 

We think that another reason for the inflammogenicity of the higher Mw PAA is that the higher Mw PAA may degrade slower than the lower Mw PAA, leading to lung damage. Considering that sustained inflammation by deposited chemicals leads to the formation of chronic and irreversible lesions, such as lung fibrosis and tumors [7,8,9,10,11], chemicals with slower clearance from the lung are thought to be an important factor in lung disorder resulting from chemicals. It has been reported that the higher-molecular-weight hyaluronic acid (HA), an organic compound like PAA, shows longer persistence in the lungs and delays mucociliary clearance to a greater extent the lower-molecular-weight HA following intratracheal instillation of HA with different molecular weights [35].

The CINC family are typical chemokines that induce and activate neutrophils and macrophages in rat lung. An increase in CINC-1 and CINC-2 concentrations in BALF [14,15,36] was also observed in an intratracheal instillation of nanoparticles with high toxicity under the same exposure dose as in the present study. The CINC-1 and CINC-2 concentrations in BALF increased due to PAA exposure in both PAA with HMW and LMW, but the difference between the inflammation induced by HMW and LMW PAA that was observed in the histopathology of the lung was not reflected in the CINC results. The results of these chemokines in the present study did not reflect the differences in the inflammation in the lung, and we considered that the mechanism of pulmonary inflammation induced by PAA may be different to the pulmonary inflammation induced by nanoparticles.

We measured HO-1 concentration in rat lung induced by PAA as an inflammation-related factor. We previously reported in a comprehensive gene analysis that the upregulation of HO-1 in lung induced by CL-PAA was higher in the group of “response to oxidative stress”, and 4.62 times stronger compared to the control group [4]. In the present study, the HO-1 protein level in the lung tissue induced by the HMW PAA increased persistently during the observation period compared with that by the LMW. We previously observed a persistent increase in the HO-1 protein level in lung tissue exposed to NiO nanoparticles with high toxicity [11], whereas, in contrast, the HO-1 in BALF increased transiently in an intratracheal instillation of titanium dioxide (TiO_2_) nanoparticles (Rutile) with low pulmonary toxicity [14]. The difference in the expression of HO-1 by nanoparticles with different toxicities reflects the difference in the inflammation of nanoparticles, and in this sense, the result of HO-1 due to the difference in molecular weight of PAA reflects the difference in lung inflammation. Regarding organic compounds, there is a report on PHMG-induced oxidative stress in which 4-hydroxynoneal as a biomarker of lipid peroxidation formed in inhaled rat lung exposed to polyhexamethylene guanidine hydrochloride [37]. These data suggest that oxidative stress is involved in PAA-induced lung disorder. It has been reported that oxidative stress makes lung injury more progressive in knockout mice of class A scavenger receptors (SR-As) [38].

Intratracheal instillation studies can be helpful for approximating the hazardous effects of inhalable chemicals, but a limitation of this study is that its exposure route was not physiological, despite the instillation of PAA of a respirable size; it is not the same as in inhalation studies. Therefore, inhalation studies also need to be performed to clarify whether exposure to PAA induces pulmonary inflammation and fibrosis.

## 4. Materials and Methods

### 4.1. Sample Polymers

HMW PAA (Poly acrylic acid 1,000,000:162-18601) (FUJIFILM Wako Pure Chemical Corporation, Osaka, Japan) and LMW PAA (Poly acrylic acid 25,000:162-18581) (FUJIFILM Wako Pure Chemical Corporation, Osaka, Japan) were used. The polymer was mixed with distilled water, and gently stirred for 40 min (Mag-Mixer MF820 or MD300, Yamato Scientific Co., Ltd., Tokyo, Japan). The weight average molecular weight (*M*_W_), the number average molecular weight (Mn) and the poly dispersity index (PDI) of the polymer and the radius of gyration (Rg) were measured by gel permeation chromatography (GPC) (a Prominence 501 system (SHOKO SCIENCE, Kanagawa, Japan) coupled with multi-angle static light scattering (MALS) detector (Dawn-Heleos-Ⅱ, Wyatt Technology Europe GmbH, Dernbach, Rheinland-Pfalz, Germany) using GF-7MHQ (Showa Denko K.K., Tokyo, Japan) with 0.1 M carbonate-bicarbonate buffer as the eluent [39,40]. The hydrodynamic diameter (Rh) was measured by dynamic light scattering (DLS) (DynaPro NanoStar, Wyatt Technology Corp., Santa Barbara, CA, USA) using the method of Stokes-Einstein relationship of Brownian motion [41]. In the molecular dispersion, the polymers were dissolved in 0.1 M carbonate–bicarbonate buffer, then the solutions were alkalized with 2N NaOH and then neutralized with 1N HCl. The secondary diameter was measured using SEM imaging [42,43].

The bulk polymer and the dispersed polymer in the solution were observed by a SEM by HITACHI S-4500 (Hitachi, Ltd., Tokyo, Japan).

The particle preparations in our experiment were tested for endotoxin by a gel clot endotoxin assay kit (Toxin Sensor^TM^) (Gen Script USA Inc., Piscataway, NJ, USA), the gel-clot methods with the sensitivity of 0.25 EU/mL, according to the manufacture’s instruction. 

### 4.2. Animals

Male Fischer 344 rats (8 weeks old) were purchased from Charles River Laboratories International, Inc. (Kanagawa, Japan) and kept for acclimatization for 4 weeks. They were raised under the same conditions as we described previously [4]. Briefly, they were accommodated in the Laboratory Animal Research Center of the University of Occupational and Environmental Health, Japan with free access to a commercial diet and water under a light/dark 12 h/12 h cycle, 20–25 °C, 40–70% humidity with ventilation 15 times/hour. All procedures and animal handling were performed according to the guidelines described in the Japanese Guide for the Care and Use of Laboratory Animals as approved by the Animal Care and Use Committee, University of Occupational and Environmental Health, Japan (animal studies ethics clearance proposal number; AE17-009).

### 4.3. Intratracheal Instillation

Rats (12 weeks old) received 0.2 mg (0.8 mg/kg BW) or 1.0 mg (4.0 mg/kg BW) of PAA suspended in 0.4 mL distilled water in single intratracheal instillations. Distilled water was administered to the control group. The accumulated exposure in workers who handle this dosage of CL-PAA was estimated, which may correspond to a period of approximately 529 days (1.45 years) of inhalation at 2.1 mg/m^3^, which is the maximum concentration of personal exposure of respirable dust in the workplace, as defined by the American Conference of Governmental Industrial Hygienists (ACGIH) (lung weight in rat: 2 g, lung weight in human: 1000 g, tidal volume in human: 0.625 L, respiratory rate: 15/min, exposure time per day: 8 h, deposition fraction: 10%).

### 4.4. Animals Following Intratracheal Instillation

Five rats each were assigned to each exposure and control group at 3 days, 1 week, 1 month, 3 months and 6 months after intratracheal instillation. Animals were sacrificed at each time point under anesthesia with isoflurane (Pfizer Japan, Tokyo, Japan) inhalation. After the measurement of body weight, dissection and sample collection was performed in the same manner as we described previously [44]. Briefly, BALF was collected from the right lungs following the removal of blood from the abdominal aorta and perfusion of the right lungs with normal saline. The third lobes of the right lungs were then stored at −80 °C and the left lungs were inflated and fixed using 10% formaldehyde under a pressure of 25 cm H_2_O. 

### 4.5. Cytospin Analysis of Inflammatory Cells and Measurement of LDH in BALF

BALF pellet and supernatant obtained after centrifugation (400× *g* at 4 °C for 15 min) were used for LDH measurement and cytospin analysis, respectively. Part of the BALF supernatant was stored at −80 °C for the measurement of chemokines. The BALF pellet was treated by the same procedure as in our previous report [44], then the number of total cells was counted by ADAM-MC (AR BROWN Co., Ltd., Tokyo, Japan). The cells splashed on a glass slide using cytospin (Cyto-Tek^®^ Centrifuge, Sakura Finetek Japan Co., Ltd., Tokyo, Japan) were stained with Diff-Quik (Sysmex CO., Kobe, Hyogo, Japan). The number of neutrophils, alveolar macrophages and lymphocytes was examined by microscopic observation (BX50, OLYMPUS, Tokyo, Japan). The released LDH activity in the BALF supernatant was measured by a Cytotoxicity Detection Kit^PLUS^ (LDH) (Roche Diagnostics GmbH, Mannheim, Nordrhein-Westfalen, Germany) according to the manufacturer’s instructions, estimating by a standard curve obtained from known concentrations of recombinant LDH from rabbit muscle (Roche Diagnostics GmbH, Mannheim, Nordrhein-Westfalen, Germany).

### 4.6. Measurement of Chemokines in BALF and HO-1 in Lung Tissue

The concentrations of CINC-1 and CINC-2 in the BALF supernatant were measured by ELISA kits, #RCN100 and #RCN200 (R&D Systems, Minneapolis, MN, USA), respectively. All measurements were performed according to the manufacturer’s instructions. The third lobes of the right lungs were homogenized, and the protein concentration of the lung homogenate supernatant was measured as we reported previously [44]. HO-1 concentration in the lung homogenate supernatant was measured by ELISA kit, ADI-EKS-810A (Enzo Life Sciences, Farmingdale, NY, USA).

### 4.7. 3D Micro-CT Imaging

For 3 of the 5 animals in each group, a 3D micro-CT scan was performed hours to days before dissection at each observation point. The X-ray 3D micro-CT system (CosmoScan GX, Rigaku Co., Tokyo, Japan) was operated under the following conditions: scanning time of 4.0 min, average whole body exposure of 161.9 mGy/scan, tube voltage of 90 kV, tube current of 88 µA, and chest CT of 60 × 40 mm field of view (FOV) (voxel matrix: 512×512×512 µm, and voxel size: 120×120×120 µm). The rats were in the prone position during scanning, and sevoflurane (Pfizer Japan, Tokyo, Japan) and oxygen were supplied through a nose cone. The images were retrospectively gated at both respiratory phases (inspiration and expiration).

### 4.8. Histopathology

Formaldehyde-fixed lung tissue was embedded in paraffin and cut to a thickness of 4μm, and then hematoxylin and eosin (HE) and Masson trichrome (MT) staining were performed. The lung inflammation and fibrosis were examined using the inflammatory cell infiltration score [44] and the Ashcroft score [45], respectively, according to previous reports [4,44]. Briefly, the inflammatory cell infiltration score was obtained by scoring the degree of inflammatory cell infiltration in lung tissue as follows: none (0), minimal (0.5), mild (1), moderate (2), or severe (3). The score was calculated by the following equation: Σ (grade × number of animals with grade). The Ashcroft score was assessed for lung fibrosis by a grade of 0 (normal lung) to 8 (most severe fibrosis), and the grades were summed up and then divided by the number of fields. The slides were evaluated for histological changes by a board-certified pathologist.

### 4.9. Statistical Analysis

Statistical analysis was performed by JMP^®^ Pro software (JMP Version 14.2.0, SAS Institute Inc., Cary, NC, USA). *p* values < 0.05 were considered statistically significant. One-way or two-way analysis of variance (ANOVA) followed by Dunnett’s tests or Turkey’s test were used appropriately in order to detect individual differences between the exposed groups and the controls, and among each observation point in the exposed groups, respectively. 

## 5. Conclusions

In the present study, we conducted intratracheal instillation of PAA with different molecular weights in rats and investigated lung inflammation and fibrosis in an observation period of 3 days to 6 months. The HMW PAA caused severe and persistent lung inflammation and fibrosis compared with the LMW. The difference in the inflammation induced by the HMW and LMW did not correspond to the difference in the expression of chemokines such as CINC-1 and CINC-2, but did correspond to the difference in HO-1 expression in the lung tissue. Increased molecular weight induced a persistence of lung disorder induced by PAA, suggesting that molecular weight is a physicochemical characteristic of PAA-induced lung disorder.

## Figures and Tables

**Table 1:** Physiochemical characterization of the polymers used in the present study.

Name		Polyacrylic Acid	Structural Formula	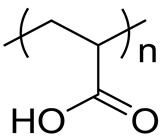
CAS number			9003-01-4	
			High molecular weight	Low molecular weight
Bulk	Purity		≦100%	≦100%
	Weight average molecular weight (*M*_W_)		598,000 g/mol	30,900 g/mol
	Number average molecular weight (Mn)		451,000 g/mol	25,800 g/mol
	Poly dispersity index (PDI)		1.33	1.20
	Cross-linking		None	None
	Appearance		Solid, white powdered	Solid, white powdered
	Odor		None	None
	Secondary diameter		2.83 µm	269 nm
Suspension	Radius of gyration (Rg)		57.5 nm	13.5 nm
	Hydrodynamic radius (Rh)		39.7 nm (Molecular dispersion)	2.78 nm (Molecular dispersion)
			217 nm (H_2_O dispersion: Suspension for instillation)	1.11 nm (H_2_O dispersion: Suspension for instillation)
	Secondary diameter		39.3 nm	53.2 nm

Secondary diameter: the particle diameter of agglomerate.

**Table 2:** Relative lung weight.

		3 Days	1 Week	1 Month	3 Months	6 Months
Relative lung weight (% ± SD)						
Control (distilled water)		0.36 ± 0.03	0.34 ± 0.01	0.32 ± 0.01	0.32 ± 0.01	0.32 ± 0.01
HMW	0.2 mg	0.50 ± 0.03 **	0.48 ± 0.02 **	0.38 ± 0.01 **	0.33 ± 0.01	0.32 ± 0.01
HMW	1.0 mg	0.73 ± 0.03 **	0.70 ± 0.05 **	0.50 ± 0.01 **	0.43 ± 0.02 **	0.39 ± 0.02 **
Control (distilled water)		0.37 ± 0.01	0.35 ± 0.03	0.33 ± 0.02	0.30 ± 0.01	0.30 ± 0.03
LMW	0.2 mg	0.42 ± 0.02 **	0.38 ± 0.02	0.34 ± 0.01	0.31 ± 0.01	0.30 ± 0.01
LMW	1.0 mg	0.54 ± 0.02 **	0.48 ± 0.02 **	0.39 ± 0.02 **	0.31 ± 0.01 *	0.30 ± 0.01

Data are presented as mean ± SE. (* *p* < 0.05, ** *p* < 0.01).

**Table 3:** Inflammatory cell counts in BALF.

		3 Days	1 Week	1 Month	3 Months	6 Months
Total cell count (×1000 cells/mL ± SD)						
Control (distilled water)		122.4 ± 36.3	137.5 ± 21.8	98.4 ± 34.5	185.0 ± 64.5	174.8 ± 59.0
HMW	0.2 mg	580.0 ± 228.9 **	449.4 ± 42.5 *	403.7 ± 42.3 **	191.7 ± 29.6	212.3 ± 74.3
HMW	1.0 mg	1043.6 ± 165.3 **	592.3 ± 189.0 **	407.3 ± 154.6 **	166.0 ± 53.2	186.5 ± 34.2
Control (distilled water)		94.5 ± 9.9	69.3 ± 14.5	80.4 ± 28.4	75.2 ± 19.6	89.4 ± 28.2
LMW	0.2 mg	342.2 ± 98.5 **	147.1 ± 27.9	88.8 ± 39.0	91.2 ± 15.3	102.9 ± 30.1
LMW	1.0 mg	1021.1 ± 116.9 **	606.0 ± 235.8 **	61.4 ± 15.3	59.4 ± 17.7	96.8 ± 21.4
Macrophage count (×1000 cells/mL ± SD)						
Control (distilled water)		121.6 ± 34.8	137.2 ± 21.8	97.4 ± 34.1	183.8 ± 63.5	172.4 ± 57.6
HMW	0.2 mg	215.5 ± 92.9	272.2 ± 41.9 *	359.9 ± 37.7 **	190.4 ± 29.4	210.6 ± 72.3
HMW	1.0 mg	411.7 ± 73.1 **	379.3 ± 114.5 **	395.0 ± 158.0 **	164.9 ± 53.3	182.6 ± 32.2
Control (distilled water)		93.0 ± 9.1	69.1 ± 14.6	79.6 ± 27.8	73.9 ± 19.4	87.9 ± 28.3
LMW	0.2 mg	168.1 ± 29.2 **	128.4 ± 22.1	87.9 ± 38.6	90.2 ± 15.3	102.2 ± 29.2
LMW	1.0 mg	255.5 ± 28.5 **	323.0 ± 137.8 **	60.6 ± 15.2	57.8 ± 16.7	95.5 ± 21.3
Neutrophil count (×1000 cells/mL ± SD)						
Control (distilled water)		1.7 ± 2.1	0.3 ± 0.4	0.4 ± 0.6	1.1 ± 1.3	1.9 ± 2.0
HMW	0.2 mg	352.2 ± 137.1 **	169.2 ± 27.2 **	33.5 ± 18.7 **	1.1 ± 0.8	1.4 ± 1.9
HMW	1.0 mg	606.1 ± 141.4 **	197.4 ± 83.0 **	5.1 ± 3.3	0.8 ± 0.6	3.9 ± 2.6
Control (distilled water)		0.7 ± 0.6	0.1 ± 0.1	0.5 ± 0.6	0.8 ± 0.8	0.9 ± 1.2
LMW	0.2 mg	171.1 ± 76.3 **	17.0 ± 9.2	0.4 ± 0.6	0.6 ± 0.8	0.4 ± 0.7
LMW	1.0 mg	739.7 ± 81.4 **	251.5 ± 93.4 **	0.7 ± 0.3	1.5 ± 1.2	0.2 ± 0.4
Percentage of neutrophil (% ± SD)						
Control (distilled water)		1.1 ± 1.3	0.2 ± 0.3	0.5 ± 0.7	0.5 ± 0.5	1.0 ± 0.7
HMW	0.2 mg	60.8 ± 4.4 **	37.8 ± 5.9 **	8.3 ± 4.5 **	0.6 ± 0.4	0.5 ± 0.7
HMW	1.0 mg	57.8 ± 6.3 **	32.8 ± 7.1 **	1.5 ± 1.3	0.5 ± 0.4	2.0 ± 1.2
Control (distilled water)		0.7 ± 0.6	0.1 ± 0.2	0.5 ± 0.5	1.0 ± 0.9	1.1 ± 1.4
LMW	0.2 mg	48.2 ± 8.7 **	11.1 ± 5.5 **	0.4 ± 0.4	0.7 ± 0.8	0.3 ± 0.4
LMW	1.0 mg	72.5 ± 2.2 **	41.6 ± 6.7 **	1.1 ± 0.5	2.2 ± 1.3	0.2 ± 0.4
Lymphocyte count (×1000 cells/mL ± SD)						
Control (distilled water)		0.0 ± 0.0	0.0 ± 0.0	0.6 ± 0.9	0.1 ± 0.3	0.4 ± 0.8
HMW	0.2 mg	1.6 ± 3.7	0.4 ± 0.8	9.9 ± 6.7 *	0.0 ± 0.0	0.3 ± 0.7
HMW	1.0 mg	7.0 ± 7.6	3.1 ± 2.6 *	7.1 ± 5.0	0.3 ± 0.4	0.0 ± 0.0
Control (distilled water)		0.7 ± 0.8	0.1 ± 0.2	0.3 ± 0.4	0.5 ± 0.5	0.5 ± 0.2
LMW	0.2 mg	2.3 ± 2.0	0.5 ± 0.7	0.4 ± 0.4	0.4 ± 0.4	0.3 ± 0.3
LMW	1.0 mg	10.8 ± 11.2	14.6 ± 12.2 *	0.1 ± 0.1	0.1 ± 0.1	1.0 ± 0.3

Data are presented as mean ± SE. (* *p* < 0.05, ** *p* < 0.01).

## Data Availability

Not applicable.

## Appendix A

**Table A1:** The results of the Ashcroft score of lung lesions.

**(A) HMW**											
**Observation Point**	**Group**	**Ashcroft Score**	**vs. 3 Days**			**vs. 1 Week**			**vs. 1 Month**		
			**Control**	**0.2 mg**	**1.0 mg**	**Control**	**0.2 mg**	**1.0 mg**	**Control**	**0.2 mg**	**1.0 mg**
3 days	Control	0.0 ± 0.0	-	N.S.	N.S.	N.S.	*p* < 0.01	*p* < 0.01	N.S.	N.S.	*p* < 0.01
	0.2 mg	0.5 ± 0.2	N.S.	-	N.S.	N.S.	N.S.	*p* < 0.01	N.S.	N.S.	*p* < 0.01
	1.0 mg	0.7 ± 0.2	N.S.	N.S.	-	N.S.	N.S.	*p* < 0.01	N.S.	N.S.	*p* < 0.01
1 week	Control	0.0 ± 0.0	N.S.	N.S.	N.S.	-	*p* < 0.01	*p* < 0.01	N.S.	N.S.	*p* < 0.01
	0.2 mg	1.0 ± 0.5	N.S.	N.S.	N.S.	N.S.	-	*p* < 0.01	*p* < 0.01	N.S.	*p* < 0.01
	1.0 mg	3.4 ± 0.9	N.S.	N.S.	N.S.	N.S.	N.S.	-	*p* < 0.01	*p* < 0.01	
1 month	Control	0.0 ± 0.0	N.S.	N.S.	N.S.	N.S.	N.S.	N.S.	-	N.S.	*p* < 0.01
	0.2 mg	0.6 ± 0.1	N.S.	N.S.	N.S.	N.S.	N.S.	N.S.	N.S.	-	*p* < 0.01
	1.0 mg	3.1 ± 0.4	N.S.	N.S.	N.S.	N.S.	N.S.	N.S.	N.S.	N.S.	-
3 months	Control	0.0 ± 0.0	N.S.	N.S.	N.S.	N.S.	N.S.	N.S.	N.S.	N.S.	N.S.
	0.2 mg	0.1 ± 0.1	N.S.	N.S.	N.S.	N.S.	N.S.	N.S.	N.S.	N.S.	N.S.
	1.0 mg	2.5 ± 0.4	N.S.	N.S.	N.S.	N.S.	N.S.	N.S.	N.S.	N.S.	N.S.
6 months	Control	0.0 ± 0.0	N.S.	N.S.	N.S.	N.S.	N.S.	N.S.	N.S.	N.S.	N.S.
	0.2 mg	0.1 ± 0.1	N.S.	N.S.	N.S.	N.S.	N.S.	N.S.	N.S.	N.S.	N.S.
	1.0 mg	2.0 ± 0.8	N.S.	N.S.	N.S.	N.S.	N.S.	N.S.	N.S.	N.S.	N.S.
**(A) HMW**											
**Observation Point**	**Group**	**Ashcroft Score**	**vs. 3 Months**				**vs. 6 Months**				
			**Control**	**0.2 mg**		**1.0 mg**	**Control**		**0.2 mg**	**1.0 mg**	
3 days	Control	0.0 ± 0.0	N.S.	N.S.		*p* < 0.01	N.S.		N.S.	*p* < 0.01	
	0.2 mg	0.5 ± 0.2	N.S.	N.S.		*p* < 0.01	N.S.		N.S.	*p* < 0.01	
	1.0 mg	0.7 ± 0.2	N.S.	N.S.		*p* < 0.01	N.S.		N.S.	*p* < 0.01	
1 week	Control	0.0 ± 0.0	N.S.	N.S.		*p* < 0.01	N.S.		N.S.	*p* < 0.01	
	0.2 mg	1.0 ± 0.5	*p* < 0.01	*p* < 0.01		*p* < 0.01	*p* < 0.01		*p* < 0.01	*p* < 0.01	
	1.0 mg	3.4 ± 0.9	*p* < 0.01	*p* < 0.01		*p* < 0.01	*p* < 0.01		*p* < 0.01	*p* < 0.01	
1 month	Control	0.0 ± 0.0	N.S.	N.S.		*p* < 0.01	N.S.		N.S.	*p* < 0.01	
	0.2 mg	0.6 ± 0.1	N.S.	N.S.		*p* < 0.01	N.S.		N.S.	*p* < 0.01	
	1.0 mg	3.1 ± 0.4	*p* < 0.01	*p* < 0.01		N.S.	*p* < 0.01		*p* < 0.01	*p* < 0.01	
3 months	Control	0.0 ± 0.0	-	N.S.		*p* < 0.01	N.S.		N.S.	*p* < 0.01	
	0.2 mg	0.1 ± 0.1	N.S.	-		*p* < 0.01	N.S.		N.S.	*p* < 0.01	
	1.0 mg	2.5 ± 0.4	N.S.	N.S.		-	*p* < 0.01		*p* < 0.01	N.S.	
6 months	Control	0.0 ± 0.0	N.S.	N.S.		N.S.	-		N.S.	*p* < 0.01	
	0.2 mg	0.1 ± 0.1	N.S.	N.S.		N.S.	N.S.		-	*p* < 0.01	
	1.0 mg	2.0 ± 0.8	N.S.	N.S.		N.S.	N.S.		N.S.	-	
**(B) LMW**											
**Observation** **Point**	**Group**	**Ashcroft Score**	**vs. 3 Days**			**vs. 1 Week**			**vs. 1 Month**		
			**Control**	**0.2 mg**	**1.0 mg**	**Control**	**0.2 mg**	**1.0 mg**	**Control**	**0.2 mg**	**1.0 mg**
3 days	Control	0.0 ± 0.0	-	N.S.	*p* < 0.01	N.S.	N.S.	*p* < 0.01	N.S.	N.S.	N.S.
	0.2 mg	0.0 ± 0.1	N.S.	-	*p* < 0.01	N.S.	N.S.	*p* < 0.01	N.S.	N.S.	N.S.
	1.0 mg	0.5 ± 0.2	N.S.	N.S.	-	*p* < 0.01	*p* < 0.01	*p* < 0.01	*p* < 0.01	*p* < 0.01	*p* < 0.01
1 week	Control	0.0 ± 0.0	N.S.	N.S.	N.S.	-	N.S.	*p* < 0.01	N.S.	N.S.	N.S.
	0.2 mg	0.1 ± 0.1	N.S.	N.S.	N.S.	N.S.	-	*p* < 0.01	N.S.	*p* < 0.01	N.S.
	1.0 mg	0.4 ± 0.2	N.S.	N.S.	N.S.	N.S.	N.S.	-	*p* < 0.01	*p* < 0.01	*p* < 0.01
1 month	Control	0.0 ± 0.0	N.S.	N.S.	N.S.	N.S.	N.S.	N.S.	-	N.S.	N.S.
	0.2 mg	0.0 ± 0.0	N.S.	N.S.	N.S.	N.S.	N.S.	N.S.	N.S.	-	N.S.
	1.0 mg	0.1 ± 0.1	N.S.	N.S.	N.S.	N.S.	N.S.	N.S.	N.S.	N.S.	-
3 months	Control	0.0 ± 0.0	N.S.	N.S.	N.S.	N.S.	N.S.	N.S.	N.S.	N.S.	N.S.
	0.2 mg	0.0 ± 0.0	N.S.	N.S.	N.S.	N.S.	N.S.	N.S.	N.S.	N.S.	N.S.
	1.0 mg	0.0 ± 0.0	N.S.	N.S.	N.S.	N.S.	N.S.	N.S.	N.S.	N.S.	N.S.
6 months	Control	0.0 ± 0.0	N.S.	N.S.	N.S.	N.S.	N.S.	N.S.	N.S.	N.S.	N.S.
	0.2 mg	0.0 ± 0.0	N.S.	N.S.	N.S.	N.S.	N.S.	N.S.	N.S.	N.S.	N.S.
	1.0 mg	0.0 ± 0.0	N.S.	N.S.	N.S.	N.S.	N.S.	N.S.	N.S.	N.S.	N.S.
**(B) LMW**											
**Observation** **Point**	**Group**	**Ashcroft Score**	**vs. 3 Months**				**vs. 6 Months**				
			**Control**	**0.2 mg**		**1.0 mg**	**Control**		**0.2 mg**	**1.0 mg**	
3 days	Control	0.0 ± 0.0	N.S.	N.S.		N.S.	N.S.		N.S.	N.S.	
	0.2 mg	0.5 ± 0.2	N.S.	N.S.		N.S.	N.S.		N.S.	N.S.	
	1.0 mg	0.7 ± 0.2	*p* < 0.01	*p* < 0.01		*p* < 0.01	*p* < 0.01		*p* < 0.01	*p* < 0.01	
1 week	Control	0.0 ± 0.0	N.S.	N.S.		N.S.	N.S.		N.S.	N.S.	
	0.2 mg	1.0 ± 0.5	N.S.	N.S.		N.S.	N.S.		N.S.	N.S.	
	1.0 mg	3.4 ± 0.9	*p* < 0.01	*p* < 0.01		*p* < 0.01	*p* < 0.01		*p* < 0.01	*p* < 0.01	
1 month	Control	0.0 ± 0.0	N.S.	N.S.		N.S.	N.S.		N.S.	N.S.	
	0.2 mg	0.6 ± 0.1	N.S.	N.S.		N.S.	N.S.		N.S.	N.S.	
	1.0 mg	3.1 ± 0.4	N.S.	N.S.		N.S.	N.S.		N.S.	N.S.	
3 months	Control	0.0 ± 0.0	-	N.S.		N.S.	N.S.		N.S.	N.S.	
	0.2 mg	0.1 ± 0.1	N.S.	-		N.S.	N.S.		N.S.	N.S.	
	1.0 mg	2.5 ± 0.4	N.S.	N.S.		-	N.S.		N.S.	N.S.	
6 months	Control	0.0 ± 0.0	N.S.	N.S.		N.S.	-		N.S.	N.S.	
	0.2 mg	0.1 ± 0.1	N.S.	N.S.		N.S.	N.S.		-	N.S.	
	1.0 mg	2.0 ± 0.8	N.S.	N.S.		N.S.	N.S.		N.S.	-	

*p* < 0.01 indicate that there is a significant difference in the scores between the two groups. N.S. indicates that there is no significant difference in the scores between the two groups.
